# Supplementary material for: Bioelectronic Nose Based on Single-Stranded DNA and Single-Walled Carbon Nanotube to Identify a Major Plant Volatile Organic Compound (p-Ethylphenol) Released by Phytophthora Cactorum Infected Strawberries
Source: Nanomaterials (Basel). 2020 Mar 7;10(3):479. doi: 10.3390/nano10030479 (PMC7153259; doi:10.3390/nano10030479)
Supplement: Supplementary file 1 [file nanomaterials-10-00479-s001.pdf]

Supporting Information

Bioelectronic Nose Based on Single-Stranded DNA and Single-Walled Carbon Nanotube to Identify a Major Plant Volatile Organic Compound (p-Ethylphenol) Released by Phytophthora Cactorum Infected Strawberries

Hui Wang [1,2,*], Yue Wang [1] , Xiaopeng Hou [2] and Benhai Xiong [1,*]

[1] State Key Laboratory of Animal Nutrition, Institute of Animal Sciences, Chinese Academy of Agricultural Sciences, Beijing 100193, China; wangyue9313@163.com (Y.W.)

[2] Research Institute of Wood Industry, Chinese Academy of Forestry, Beijing 100091, China; houxiaopeng_lunwen@163.com (X.H.)

* Correspondence: wanghui_lunwen@163.com or wanghuilunwen@cau.edu.cn (H.W.); xiongbenhai@caas.cn (B.X.); Tel.: +86-010-62811680 (B.X.)

Received: 05 February 2020; Accepted: 03 March 2020; Published: date

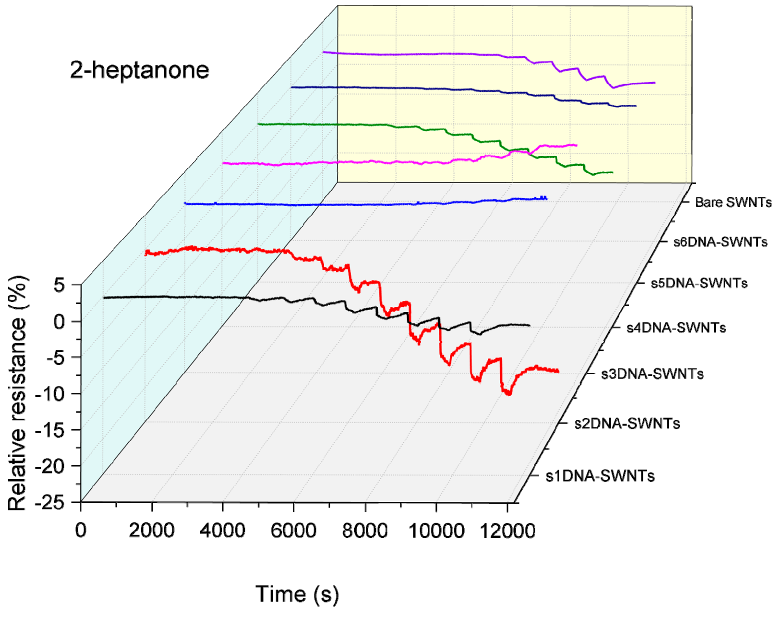

Figure S1. The relative resistance of different ssDNA-SWNTs towards different concentrations of 2-heptanone varying from 0.25% to 100%.

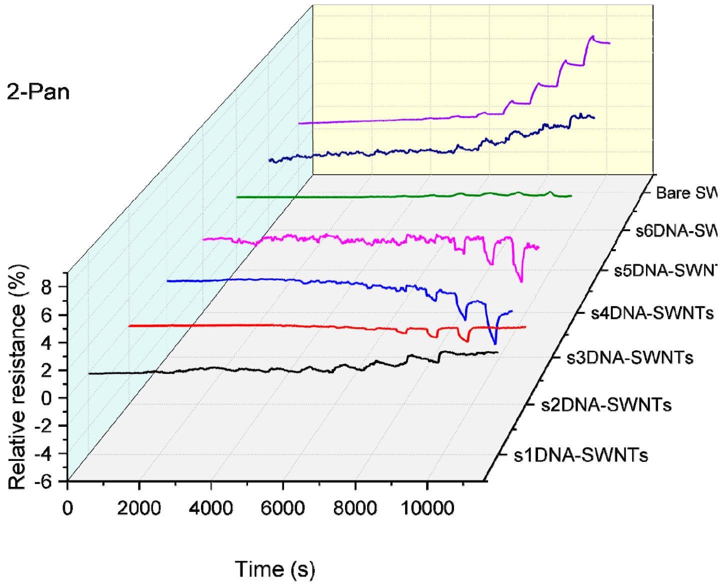

Figure S2. The relative resistance of different ssDNA-SWNTs towards different concentrations of 2-pentanone varying from 0.25% to 100%.

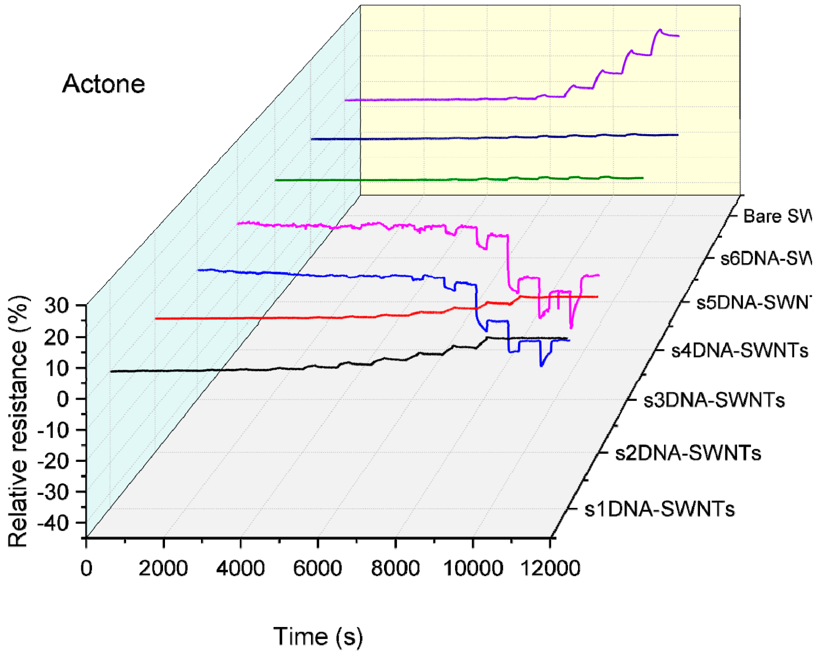

Figure S3. The relative resistance of different ssDNA-SWNTs towards different concentrations of actone varying from 0.25% to 100%.

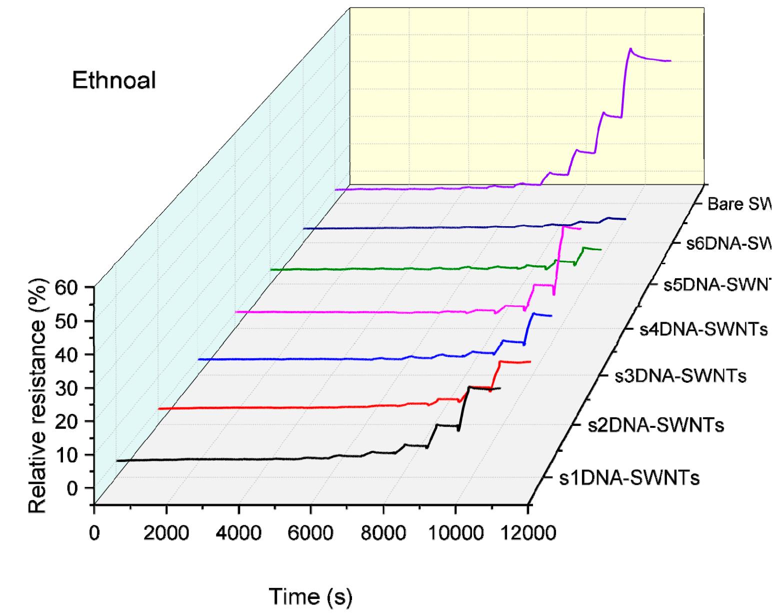

Figure S4. The relative resistance of different ssDNA-SWNTs towards different concentrations of ethnoal varying from 0.25% to 100%.

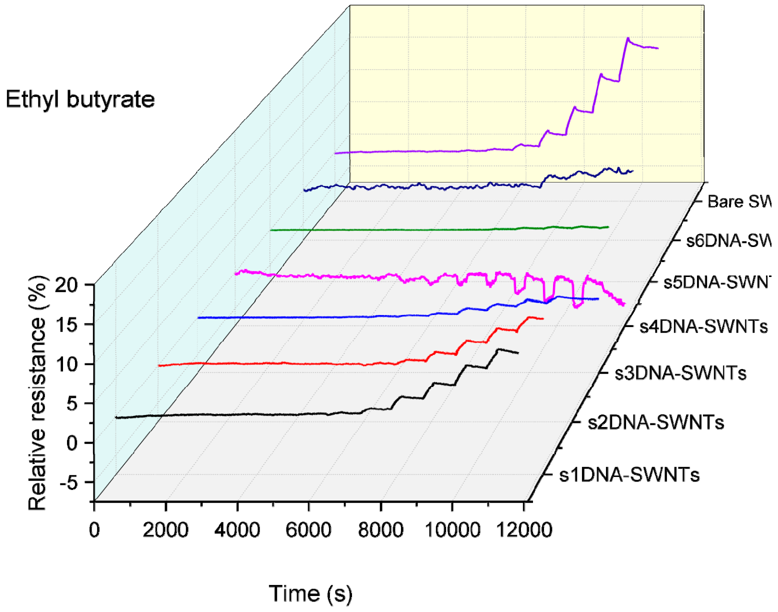

Figure S5. The relative resistance of different ssDNA-SWNTs towards different concentrations of ethyl butanoate varying from 0.25% to 100%.

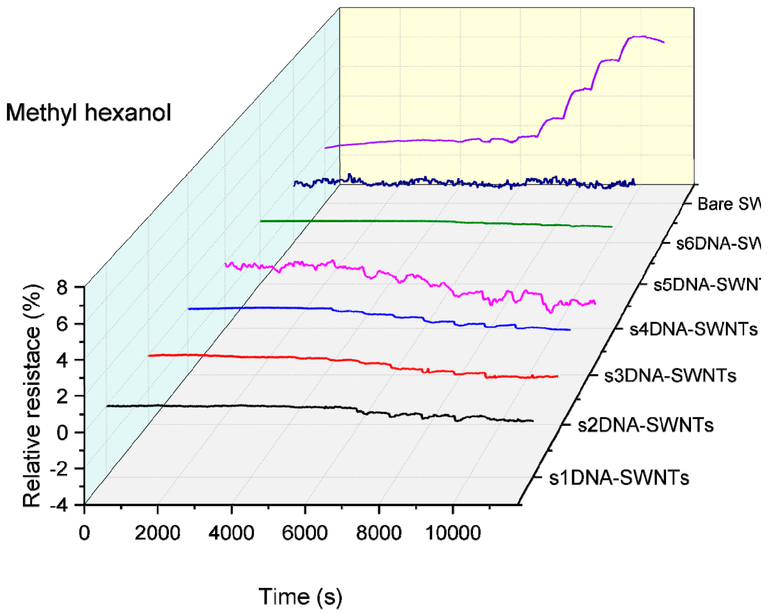

Figure S6. The relative resistance of different ssDNA-SWNTs towards different concentrations of methyl hexanoate varying from 0.25% to 100%.

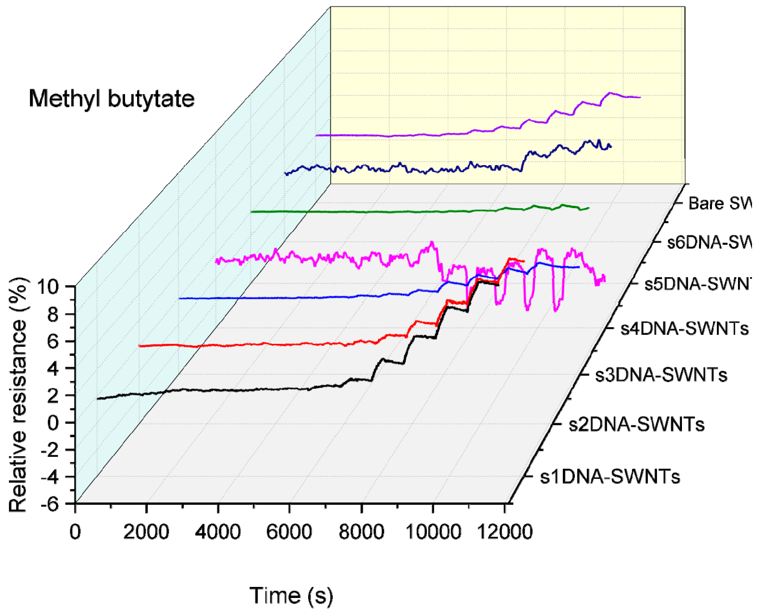

Figure S7. The relative resistance of different ssDNA-SWNTs towards different concentrations of methyl butanoate varying from 0.25% to 100%.

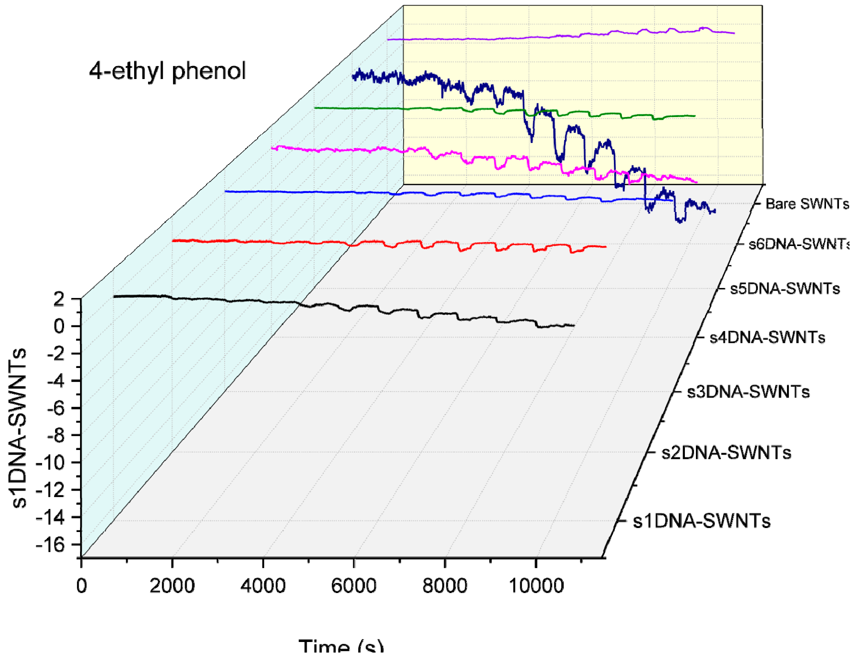

Figure S8. The relative resistance of different ssDNA-SWNTs towards different concentrations of 4-ethyl phenol varying from 0.25% to 100%.
